# Atlas of Methods of Clinical Investigation, with an Epitome of Clinical Diagnosis and of Special Pathology and Treatment of Internal Diseases

**Published:** 1899-06

**Authors:** 


**Atlas of Methods of Clinical Investigation, with an Epitome of Clinical Diagnosis and of Special Pathology and Treatment of Internal Diseases.** By Dr. Christfried Jakob. Edited by Augustus A. Eshner, M.D. London: The Rebman Publishing Co. (Ltd.). 1898.

The earlier part of this book consists of 68 coloured plates, containing 182 illustrations, dealing respectively with clinical microscopy, chemical colour-reactions, the normal projection of the viscera upon the surface of the body, and their topography as determined by percussion, together with diagrammatic representations of diseases of the chest and abdomen. The plates are accompanied by descriptive letter-press, the object being to represent in a graphic manner the results of the clinical investigation of diseases by all the above methods. The number of the plates is so large that the microscopy of the blood, urine, sputum, gastric and intestinal contents is fully illustrated; and they are uniformly good, so that, besides being useful to the practitioner for reference, they will doubtless greatly aid the student. The representations of the colour-reactions of the various chemical tests in clinical use will also be helpful. The diagrammatic pictures of abdominal and thoracic affections are taken from typical cases, of which a short abstract faces each plate.

The next portion of the book describes the method to be followed for the complete examination of a patient, and is followed by a detailed account of the clinical investigation of the systems of the body; of the blood and secretions, and of the chief bacteriological methods of use for direct clinical purposes, together with a short description of the normal state of each system and the chief pathological deviations from it.

Lastly, there is an epitome of special pathology and treatment. This is not so satisfactory as the rest of the book, as it attempts the impossible task of describing satisfactorily in brief abstract the symptoms and signs of disease. This is, however, a small part of the book, which for the rest is excellent, and we can recommend it as a very useful clinical manual of convenient size, and remarkable for the abundance and practical usefulness and beauty of its illustrations.

